# Inhibition of Apoptosis Signal–Regulating Kinase 1 Reduces Myocardial Ischemia–Reperfusion Injury in the Mouse

**DOI:** 10.1161/JAHA.112.002360

**Published:** 2012-10-25

**Authors:** Stefano Toldo, David G. Breckenridge, Eleonora Mezzaroma, Benjamin W. Tassell, John Shryock, Harsha Kannan, Dillon Phan, Grant Budas, Daniela Farkas, Edward Lesnefsky, Norbert Voelkel, Antonio Abbate

**Affiliations:** VCU Pauley Heart Center, Virginia Commonwealth University, Richmond, VA (S.T., H.K., E.L., A.A.); Victoria Johnson Research Laboratories, Virginia Commonwealth University, Richmond, VA (S.T., E.M., B.W.V.T., H.K., D.F., N.V., A.A.); School of Pharmacy, Virginia Commonwealth University, Richmond, VA (E.M., B.W.V.T.); Department of Physiology and Biophysics, Virginia Commonwealth University, Richmond, VA (E.L., A.A.); Department of Biochemistry, Virginia Commonwealth University, Richmond, VA (E.L.); Gilead Sciences, Foster City, CA (D.G.B., J.S., D.P., G.B.)

**Keywords:** apoptosis, inhibitors, ischemia, remodeling, reperfusion

## Abstract

**Background:**

Despite the clear advantages of reperfusion in acute myocardial infarction, part of the myocardium is injured during reperfusion by reactive oxygen species. Reactive oxygen species activate apoptosis signal–regulating kinase-1, a key mediator in cell death. We hypothesized that inhibition of apoptosis signal–regulating kinase-1 at the time of reperfusion would protect the heart from ischemia–reperfusion injury.

**Methods and Results:**

Male CD1 mice underwent transient coronary artery ligation (30 minutes) followed by reperfusion or underwent sham surgery (n=10 to 12 per group). A selective small-molecule inhibitor of apoptosis signal–regulating kinase-1 (GS-459679) was given immediately after reperfusion (10 or 30 mg/kg IP). Infarct size was measured early (at 24 hours, in a subgroup of mice) by triphenyl tetrazolium chloride staining and late (at 7 days) by Masson's trichrome staining for fibrosis. Apoptosis was assessed by measurement of caspase-3 activity and by determination of DNA fragmentation in cardiomyocytes bordering the infarct. Transthoracic echocardiography was performed before surgery and then at 24 hours and 7 days later. Treatment with GS-459679 at reperfusion led to a significant dose-related reduction in infarct size (31% for 10 mg/kg [*P*<0.001 versus vehicle] and 60% for 30 mg/kg [*P*<0.001 versus vehicle]), inhibition of apoptotic cell death, and preservation of left ventricular dimension and systolic function at both 24 hours and 7 days.

**Conclusions:**

Inhibition of apoptosis signal–regulating kinase-1 at the time of reperfusion limits infarct size and preserves left ventricular function in a model of acute myocardial infarction in the mouse.

## Introduction

Prompt reperfusion is the primary objective in the treatment of acute myocardial infarction (AMI).^1^ Despite the clear advantages of reperfusion in AMI, part of the myocardium is injured during reperfusion, and this limits the benefits of reperfusion.^2^ The amount of myocardium lost during AMI, infarct size, is one of the strongest clinical predictors of long-term outcome,^3^ and therefore optimization of reperfusion strategies to limit the infarct size is essential.

Experimental studies have indeed shown that reperfusion itself initiates a process by which part of the salvageable myocardium fails to be rescued.^2^ Oxygen depletion during ischemia inhibits mitochondrial function, which leads to a surge of reactive oxygen species (ROS) during reperfusion.^2,4^ ROS are toxic to the cell.^5^ Among the different effects of ROS, they activate apoptosis signal–regulating kinase-1 (ASK1), a newly recognized key mediator in cell death.^6^ Active, phosphorylated ASK1 functions as a signaling node for many mitogen-activated protein kinase (MAPK) kinases, primarily MAPK kinase 4 and MAPK kinase 6, which in turn activate p38MAPK and c-Jun N-terminal kinase, which ultimately are involved in cell survival and death.^6^ In a model of AMI due to ischemia–reperfusion, mice with genetic deletion of ASK1 (ASK1-knockout mice) had smaller infarct size, whereas ASK1 transgenic mice overexpressing active ASK1 had larger infarcts.^7,8^ Moreover, ASK1-knockout mice are protected from degenerative tissue changes in models of chronic heart disease.^9^

In the present study, we use a small-molecule inhibitor of ASK1 (GS-459679) developed at Gilead Sciences (Foster City, CA) to prevent reperfusion-induced ASK1 activation and to reduce infarct size in a mouse model of AMI due to ischemia–reperfusion.

## Methods

### Experimental AMI Model

**Figure 1.**
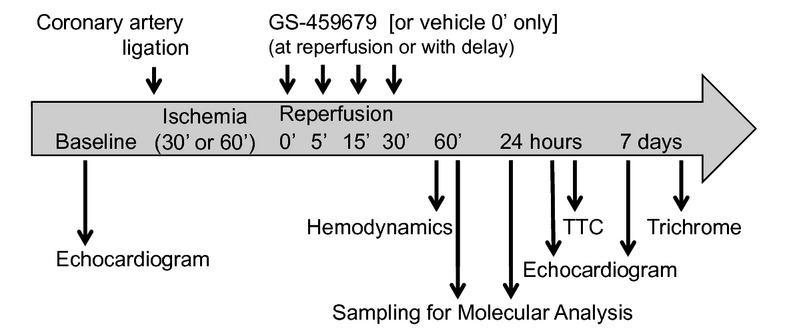
The figure shows the timeline of the study. Eight treatment groups of mice were used (total mice, N=120): (1) sham (surgery without ischemia and reperfusion) at 60 min, 24 h, or 7 d (n=4 per each time point); (2) vehicle ischemia and reperfusion (0.1 mL vehicle solution at time of reperfusion) for 60 min, for 24 h for TTC and molecular analysis, or for 7 d (n=6 per each time point); (3) ASK1 inhibitor, GS-459679, single injection at 24 h for TTC (10 mg/kg in 0.1 mL vehicle solution; n=6); (4) GS-459679 single injection (30 mg/kg in 0.1 mL vehicle solution) at 60 min, at 24 h for TTC and molecular analysis, or at 7 d (n=6 per each time point); (5) GS-459679 30 mg/kg or vehicle BID for 7 d (n=6 per treatment arm); (6) delayed administration of GS-459679 30 mg/kg at 5, 15, and 30 min after reperfusion (n=6 per treatment arm); (7) GS-459679 30 mg/kg and vehicle single injection given at reperfusion after 60 min of ischemia (n=6 per treatment arm); and (8) permanent ligation (no reperfusion) treated with GS-459679 30 mg/kg or vehicle solution BID for 7 d (n=6 per treatment arm). TTC indicates triphenyl tetrazolium chloride staining for infarct size; BID, *bis in die*, from the Latin for twice daily.

Adult out-bred male CD1 mice (8 to 12 weeks of age) were supplied by Harlan Sprague Dawley (Indianapolis, IN). The experiments were conducted under the *Guide for the Care and Use of Laboratory Animals* published by National Institutes of Health (No. 85-23, revised 1996). The study protocol was approved by the Virginia Commonwealth University Institutional Animal Care and Use Committee. Experimental AMI was induced by transient myocardial ischemia for 30 minutes and was followed by reperfusion, as described previously.^10^ Briefly, mice were orotracheally intubated under anesthesia (pentobarbital 70 mg/kg), were placed in the right lateral decubitus position, and then were subjected to left thoracotomy, pericardiectomy, and ligation of the proximal left coronary artery. The artery was released after 30 minutes of ischemia, before closure of the thorax. The mice surviving surgery were assigned randomly to the different treatment groups (n=6 to 15 per group). Two additional groups of mice (n=6 per group) underwent coronary artery ligation for 60 minutes instead of 30 minutes to induce even greater myocardial injury, whereas 2 other groups underwent ischemia without reperfusion. Sham operations were performed in which animals underwent the same surgical procedure without coronary artery ligation (n=4 to 8 per group). After surgery, buprenorfine (0.05 mg/kg) was given to the mice every 12 hours for 3 days. A timeline of the study is shown in Figure 1.

### Treatment

The ASK1 inhibitor used in this study, GS-459679, is a highly potent and selective inhibitor of ASK1 developed at Gilead Sciences (Foster City, CA).^11,12^ The compound was diluted in a polyethylene glycol-300 (Sigma-Aldrich, Saint Louis, MO) 45% water solution (pH 2.2) and was administered intraperitoneally (final volume 0.1 mL). Mice were assigned randomly to treatment with GS-459679 (10 or 30 mg/kg in 0.1 mL of vehicle solution) or with a matching volume of vehicle given as a single dose intraperitoneally at different time intervals after reperfusion (0, 5, 15, and 30 minutes). An additional group of mice was treated with 0.1 mL of NaCl 0.9% as an additional control, but because the data for vehicle treatment were not significantly different from the data for NaCl treatment, only the results of vehicle treatment are reported throughout the present article. The different treatment groups are described in the legend of Figure 1.

### Caspase-3 Activation

An additional subset of mice were euthanized 1 hour after reperfusion (n=5 per treatment group). The heart was removed as described above. The tissue activity of caspase-3, a key mediator of apoptosis, was determined by cleavage of a fluorogenic substrate (CaspACE, Promega, Madison, WI). After homogenization with RIPA buffer (Sigma-Aldrich) containing a cocktail of protease inhibitors (Sigma-Aldrich) and after centrifugation at 16 000 rpm for 20 minutes, 75 μg of protein from each sample was used for the assay, according to the supplier's instructions. Fluorescence was measured after 60 minutes, was expressed as arbitrary fluorescence units produced by 1 μg of sample per minute (fluorescence/μg per minute), and was calculated as fold change compared with the caspase-3 activity in homogenates of the hearts of sham-operated mice.

### TUNEL Assay

Another subset of mice (n=6 per group) were euthanized 24 hours after reperfusion, and their hearts were explanted and fixed in formalin 10% for at least 48 hours. A transverse section of the median third of the heart was dissected, embedded in paraffin, and cut into 5-μm-thick slides. After the antigen retrieval (Citrate Buffer 0.01 mol/L pH 6.0), terminal deoxynucleotidyl transferase dUTP nick-end labeling (TUNEL) staining was performed with the in situ cell death detection kit (Fluorescein [green], Roche Diagnostic, Indianapolis, IN), followed by incubation of a monoclonal antibody raised against cardiac actin ([1:200] Sigma-Aldrich, Saint Louis, MO) and an Alexa Fluor 594 (red) secondary antibody ([1:100] donkey anti-mouse) to quantify the TUNEL-positive cardiomyocytes. Counterstaining was performed with DAPI (6-diamidino-2-phenylinidole [1:20.000], Life Technologies, Grand Island, NY), and the slides were covered with Slow Antifade (Life Technologies). The images were acquired with an IX70 microscope and MagnaFire 1.1 software (Olympus, Center Valley, PA) with a 20× objective. Color composite images were generated and quantified with ImageJ software (National Institutes of Health, http://imagej.nih.gov/ij/, 1997–2011). Five random fields from each sample were acquired in the peri-infarct area, defined as the zone bordering the infarct where viable myocardium was prevalent.^13^ The apoptotic rate was the number of apoptotic cardiomyocytes divided by all cardiomyocytes in the field, expressed as a percentage.^13^

### Infarct Size Assessment

A subset of mice (n=4 to 6 per treatment group) underwent AMI surgery and echocardiography at 24 hours, followed by immediate euthanization for measurement of infarct size by triphenyl tetrazolium chloride (Sigma-Aldrich) staining of viable myocardium.^10^ Briefly, the heart was removed quickly after euthanization and was mounted on a Langendorff apparatus. The coronary arteries were perfused with 0.9% NaCl containing 2.5 mmol/L CaCl_2_. After the blood was washed out, ≍2 mL of 1% Evans blue dye (Sigma-Aldrich) was injected as a bolus into the aorta until most of the heart turned blue. The heart then was perfused with normal saline solution to wash out the excess Evans blue. Finally, the heart was removed, frozen, and cut into 5 to 8 transverse slices, from apex to base, of equal thickness (≍1 mm). The slices were incubated in a 1% triphenyl tetrazolium chloride isotonic phosphate buffer (pH 7.4) at room temperature for 30 minutes. The areas of infarcted tissue, the risk zone, and the whole left ventricle (LV) were determined by computer morphometry in Image-Pro Plus 6.0 software (Media Cybernetics, Silver Spring, MD).

Another subset of mice (n=4 to 6 per group) were euthanized at 7 days, and their hearts were explanted, fixed in formalin, and sliced as described earlier. Tissue slides were stained with Masson's trichrome (Sigma-Aldrich) as previously described.^10^ The areas of fibrosis and the whole LV were determined by computer morphometry in Image-Pro Plus 6.0 software.

### Echocardiography

All mice underwent transthoracic echocardiography at baseline (before surgery) and at 1 and 7 days after surgery (before euthanization). Echocardiography was performed with the Vevo770 imaging system (VisualSonics Inc, Toronto, Ontario, Canada) and with a 30-MHz probe measuring the LV end-diastolic and end-systolic diameters at M mode, as described previously,^10,14^ and according to the American Society of Echocardiography recommendations.^15^ LV fractional shortening and LV ejection fraction were calculated.^15^ Bidimensional echocardiography was used to measure infarct size as the number of akinetic or dyskinetic segments according to a 16-segment map in a mouse after AMI, as previously described.^16,17^ The investigator performing and reading the echocardiogram was blinded to the treatment allocation.

### Hemodynamic Measurements

In an additional subgroup of mice (n=4 per each group), the LV apex was punctured 1 hour after reperfusion. A Millar catheter connected to a pressure transducer (Millar Instruments, Houston, TX) was inserted to measure LV peak systolic pressure and heart rate.

### Statistics

Data are reported as mean and standard error of the mean. Calculations were completed in the SPSS 15.0 package for Windows (SPSS, Chicago, IL). In all analyses for which baseline and follow-up data were available, we used analysis of variance for repeated measures to measure the time×group interaction and to compare each of the different groups with vehicle, followed by Bonferroni correction for multiple groups. For those analyses for which paired data were not available, an analysis of variance was used to compare different groups with post hoc Student *t* test and Bonferroni correction. Corrected *P* values <0.05 were considered significant.

## Results

### ASK1 Inhibition Inhibits Caspase-3 Activation and Reduces DNA Damage

**Figure 2.**
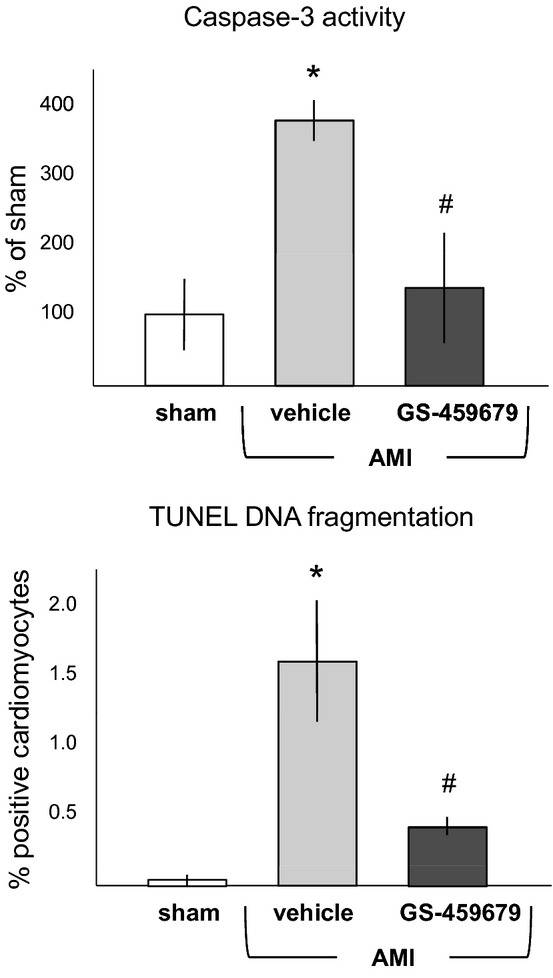
Administration of the ASK1 inhibitor GS-459679, given at reperfusion after 30 min of myocardial ischemia, inhibited caspase-3 in the heart tissue measured 60 min after reperfusion (top, *P<0.001 vs sham; #*P*<0.01 vs vehicle) and reduced apoptotic cardiomyocytes measured as TUNEL in the area bordering the infarcted myocardium 24 h after reperfusion (bottom, *P<0.001 vs sham; #*P*<0.05 vs vehicle). n=4 to 6 per group. AMI indicates acute myocardial infarction.

Caspase-3 activation is a key in the apoptotic and also necrotic cell death cascade.^18^ Treatment with GS-459679 significantly inhibited caspase-3 activity in the heart after ischemia and 60 minutes of reperfusion (Figure 2A). To confirm the attenuation of cell death, we analyzed the fragmentation of the DNA in the area bordering the infarct after ischemia and 24 hours of reperfusion by using the TUNEL assay. Consistent with the caspase-3 data, DNA fragmentation in the cardiomyocytes' nuclei was significantly reduced by ASK1 inhibition (Figure 2B).

### Infarct-Sparing Effects of ASK1 Inhibition

**Figure 3.**
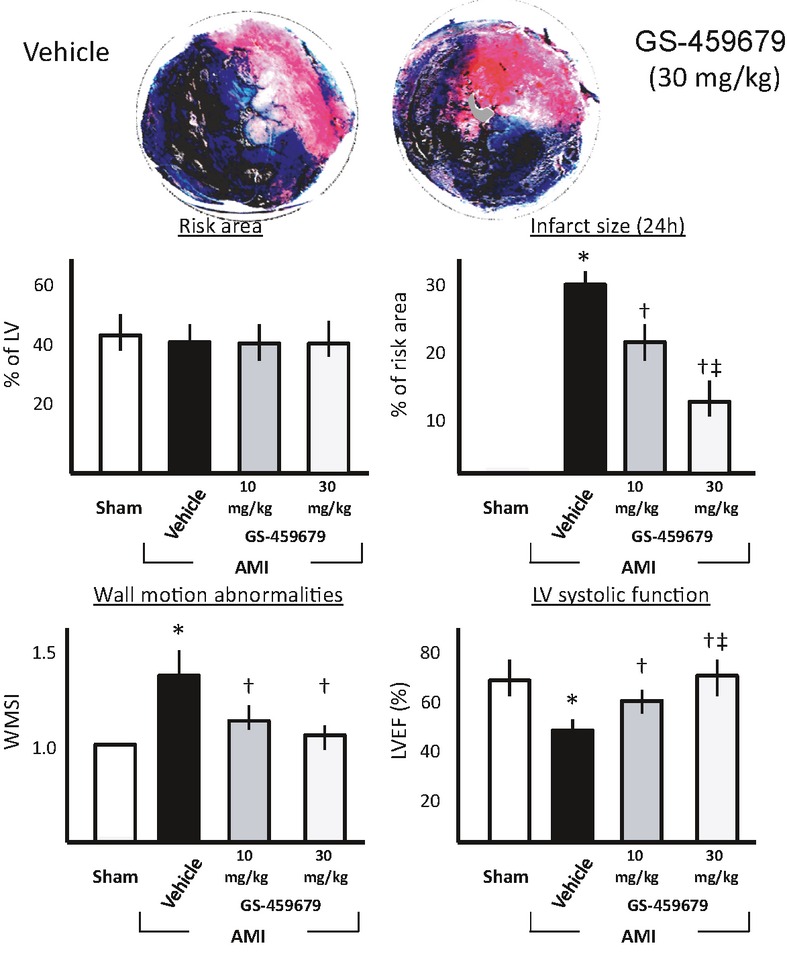
Representative images of Evans blue/triphenyl tetrazolium chloride staining of the hearts are shown in the top panels: Blue marks the nonrisk area of the heart, and the remaining is the risk area, which appears red if viable and white if infarcted. Administration of the ASK1 inhibitor GS-459679 at reperfusion led to a dose-dependent reduction in infarct size (middle panels) and preservation of contractile function (bottom panels). *P<0.001 vs sham; †*P*<0.001 vs vehicle-treated AMI; ‡*P*<0.001 vs 10 mg/kg, n=4 to 6 per group. AMI indicates acute myocardial infarction; LV, left ventricle; WMSI, wall motion score index; and LVEF, left ventricular ejection fraction.

Administration of GS-459679 at reperfusion after 30 minutes of myocardial ischemia led to a dose-dependent reduction in infarct size, measured as percent of area at risk at 24 hours (Figure 3). The reduction in infarct size was paralleled by an improvement in wall motion score indices and global LV systolic function (Figure 3). GS-459679 had no significant effect on hemodynamic parameters, such as LV systolic pressure and heart rate (Table 1). When a more severe model of ischemic injury was used, with a duration of ischemia of 60 minutes (instead of 30 minutes), the GS-459679 administered at reperfusion led to a similar degree of reduction in infarct size (19±3% of the area at risk for GS-459679 versus 32±5% for the vehicle, *P*<0.01).

**Table 1.** Gross and Hemodynamic Data

		Myocardial Infarction	

Group	Sham	GS-459679	Vehicle
Age, wk	10±1	10±1	10±1

Weight, g	31±1	33±1	32±1

LVSP, mm Hg	101±3	77±7*	78±3*

Heart rate, bpm	397±13	408±42	384±18

LVSP indicates peak left ventricular systolic pressure.

* *P*<0.001 vs sham.

### Effects of ASK1 Inhibition on Cardiac Remodeling

**Figure 4.**
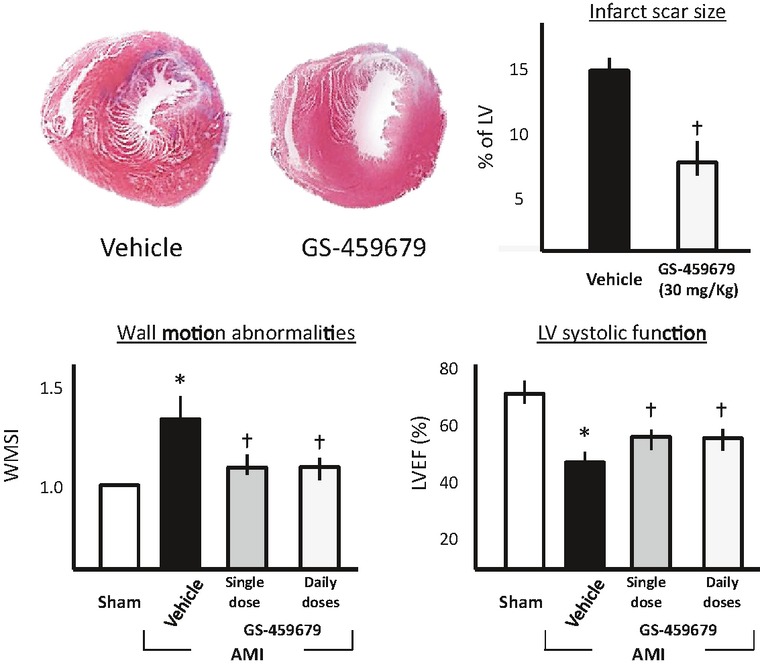
Representative images of heart sections stained with Masson's trichrome are shown: The fibrotic scar appears blue, and the viable myocardium is red. Administration of the ASK1 inhibitor (GS-459679, 30 mg/kg) at reperfusion led to a reduction in infarct scar size measured at 7 d (top panels) and preservation of contractile function (bottom panels). The administration of multiple daily doses compared with a single dose at reperfusion yielded no additional benefit. *P<0.001 vs sham; †*P*<0.001 vs vehicle-treated AMI; n=4 to 6 per group. AMI indicates acute myocardial infarction; LV, left ventricle; WMSI, wall motion score index; and LVEF, left ventricular ejection fraction.

The infarct-sparing effects of ASK1 inhibition also were evident when infarct scar size was measured 7 days after surgery, parallel with preservation of regional wall motion and global systolic function (Figure 4). To determine whether supplementation of the initial dose of GS-459679 with daily doses for 7 days provided any additional benefit, we compared infarct scar size, wall motion score index, and LV ejection fraction in the 2 groups of mice and found no significant differences between the 2 groups (Figure 4).

### Treatment Delay and Infarct-Sparing Effects

**Figure 5.**
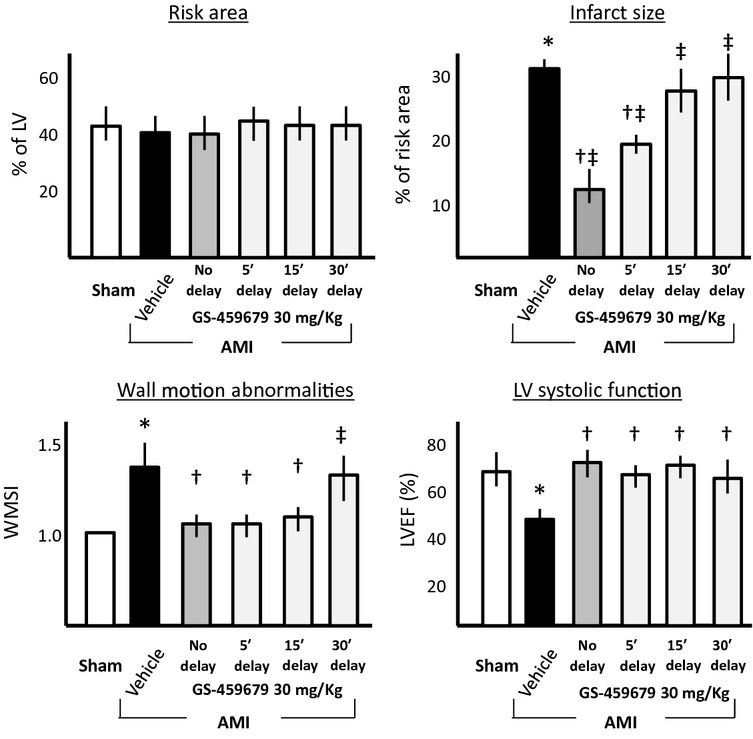
Administration of the ASK1 inhibitor (GS-459679, 30 mg/kg) with a delay at reperfusion led to a time-dependent loss of effect on infarct size (top panels) and regional wall motion abnormalities (bottom left panel), whereas global LV systolic function also was preserved with a 30-min delay (bottom right panel). *P<0.001 vs sham; †*P*<0.001 vs vehicle-treated AMI; ‡*P*<0.001 vs no delay. n=4 to 6 per group. AMI indicates acute myocardial infarction; LV, left ventricle; WMSI, wall motion score index; and LVEF, left ventricular ejection fraction.

To simulate a clinically relevant scenario in which drug treatment might occur with some delay after reperfusion, we compared treatment with GS-459679 without delay and treatment of other groups in which the inhibitor was given after 5, 15, or 30 minutes of delay. A time-dependent effect of delay on infarct size, measured as viable myocardium at 24 hours, was evident, with the drug failing to significantly reduce infarct size when it was administered after 15 or 30 minutes of delay (Figure 5). Accordingly, the 30-minute-delay group had no benefit in terms of regional wall motion at 24 hours, whereas 5- and 15-minute delays preserved regional wall motion (Figure 5). The beneficial effects on global LV systolic function at 24 hours were maintained independent of treatment delays (Figure 5).

### ASK1 Inhibition in Nonreperfused Myocardial Infarction

**Figure 6.**
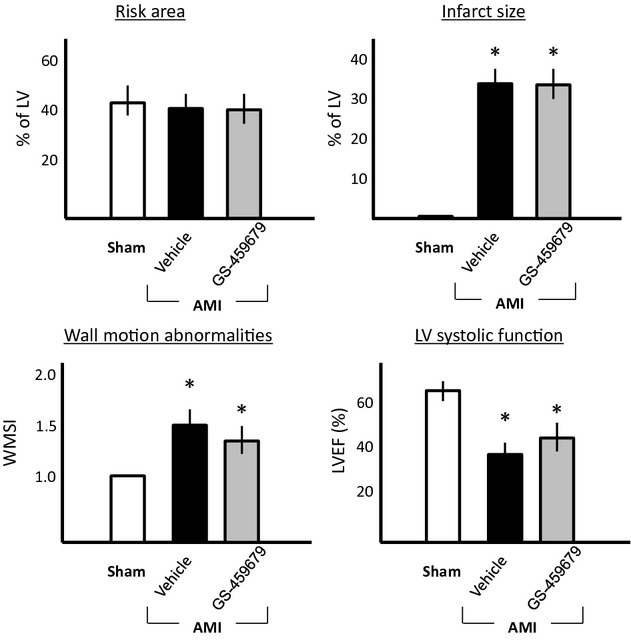
Administration of the ASK1 inhibitor (GS-459679, 30 mg/kg) after the onset of ischemia in a model of AMI without reperfusion did not affect infarct size (top panels) or regional/global systolic function (bottom panels). *P<0.001 vs sham. n=4 to 8 per group. AMI indicates acute myocardial infarction; LV, left ventricle; WMSI, wall motion score index; and LVEF, left ventricular ejection fraction.

Finally, we examined the effects of GS-459679 given at onset of ischemia in a model of severe ischemic injury due to lack of reperfusion leading to a large anterior wall AMI. In this model of severe ischemia without reperfusion, administration of GS-459679 had no significant effects on infarct size, regional wall motion, and global systolic function (Figure 6).

## Discussion

The treatment of AMI centers on prompt reperfusion.^1^ Although the benefits of reperfusion are unquestioned, some of the benefits can be lost because of injury occurring in the myocardium during the early phases of reperfusion.^2^ The present study shows for the first time that inhibition of ASK1 during AMI through the use of a pharmacological inhibitor optimizes the benefits of reperfusion by limiting reperfusion-mediated cellular injury in an in vivo model of regional transient myocardial ischemia. ASK1 is a ROS-sensitive molecular mediator that in response to stress leads to activation of an apoptotic cell death program.^6,19^ We have shown that the ASK1 pharmacological inhibitor (GS-459679), when administered at reperfusion in a clinically significant manner, rescued myocardium at risk of death. The infarct-sparing effect of the ASK1 inhibitor was associated with preservation of functional myocardium. The effects of the ASK1 inhibitor also were linked selectively with reperfusion: Indeed, if the drug was administered late after reperfusion, the infarct-sparing effects were lost, and if the drug was administered in a model of severe ischemia without reperfusion, the infarct-sparing effect was not observed. Therefore, the benefits on LV ejection fraction observed with the delayed administration of the ASK1 inhibitor seem to be independent of infarct-sparing effect. However, the mechanisms of such benefits at the moment are unclear and might be explained by a subtle infarct-sparing effect undetected with our technique, by a reduction of caspase-3 activation or apoptotic cell death in the area bordering the infarct or in the remote myocardium, or by direct involvement of ASK1 in determining cardiac contractility.

The protective effects of ASK1 inhibition are highly consistent with genetic models of ASK1 deletion. Watanabe et al^7^ showed a >50% reduction in infarct size in mice lacking ASK1 (ASK1-knockout mice) subjected to ischemia–reperfusion with the same model of regional myocardial ischemia. Liu and colleagues^8^ confirmed the key role of ASK1 in myocardial ischemia–reperfusion injury by studying mice overexpressing ASK1 selectively in the heart 3 weeks after birth (to eliminate developmental effects of ASK1), which had a 2-fold increase in infarct size after 60 minutes of regional myocardial ischemia and reperfusion. Interestingly, cardiac overexpression of ASK1 in the absence of injury (ie, ischemia or pressure overload) had no noticeable effects in mice. This is in agreement with the role of ASK1 as a cellular sensor that is activated in case of “danger” (ie, ROS surge), rather than a simple mediator of injury. ASK1 is a serine/threonine protein kinase that is expressed ubiquitously and regulates both p38 and c-Jun N-terminal kinase pathways.^6^ ASK1 is bound directly to thioredoxin-1 through the N-terminal regulatory domain of ASK1.^6^ Upon ROS generation, thioredoxin-1 is oxidized, and it dissociates from ASK1, which leads to ASK1 autoactivation through phosphorylation of Thr845. A mild surge in ROS is seen in response to the activation of many G-protein–coupled receptors in response to neurohormonal agonists like norepinephrine, angiotensin, endothelin, and others. In conditions of mild activation, ASK1 is believed to play a role in cell homeostasis and tropism.^6^ Accordingly, in chronic models of heart failure due to increased afterload (as in transverse aortic constriction^8,9^) or altered regional mechanical stress (as in nonreperfused AMI^9^), the effects of increased ASK1 become apparent only weeks after the insult, when cardiac decompensation leads to intense neurohormonal activation and markedly increased ROS generation, and the protection of ASK1 deletion against adverse cardiac remodeling is evident 2 to 4 weeks after transverse aortic constriction or nonreperfused AMI.^8,9^ A similar pattern is seen in the cardiomyopathic hamster model, in which intracoronary adenovirus transfection of a dominant-negative ASK1 mutant gene improved the heart failure phenotype.^20^ On the other hand, ASK1 activation is maximal in response to a major stress, leading to a significant ROS surge during ischemia and reperfusion, and is thought to contribute to cell death.^6^ Accordingly, ASK1-knockout mice are immediately protected from ischemia–reperfusion injury,^7^ whereas ASK1-overexpressing mice have larger injury.^8^ Our data are in agreement with a rapid activation of ASK1 during reperfusion. The signaling downstream of ASK1 is rather complex because ASK1 is an apical kinase in MAPK signaling. ASK1 seems to be linked more closely with death and survival pathways than with a hypertrophy response.^9^ Indeed, ASK1 is linked intimately with the apoptotic cascade, as the name suggests. Watanabe et al^7^ have, however, suggested that preferential inhibition of nonapoptotic cardiomyocyte cell death occurred in ASK1-knockout mice. Although previous reports showed an involvement of ASK1 in AMI, the novelty of the present study resides in the proof of ASK1 inhibition as a viable target for pharmacological intervention after AMI. The ASK1 inhibitor GS-459679 significantly reduced infarct size measured with triphenyl tetrazolium chloride and thus limited cell death. Our data also suggest a key role of ASK1 in apoptosis, inasmuch as the significant increases in caspase-3 activity in the heart 60 minutes after reperfusion and in TUNEL-positive cardiomyocytes after 24 hours were inhibited by the ASK1 inhibitor. Surprisingly, no increase in caspase-3 during myocardial ischemia and reperfusion was observed in the study by Watanabe et al.^7^ Although both apoptosis and pyroptosis (inflammatory cell death) are activated after myocardial ischemia,^21^ the early effects of ASK1 inhibition suggest this therapeutic approach to be most effective immediately after reperfusion, whereas antiinflammatory therapies (ie, interleukin-1 blockade) can be used later in the course, as inflammation occurs later during cardiac injury because of the tissue-driven sterile inflammation.^14,21,22^ The present study was designed with the goal of developing a translational program, and the use of ASK1 inhibitor after ischemia and without pretreatment is a clear example.

In conclusion, we validated ASK1 as a viable target for intervention to reduce ischemia–reperfusion injury in the heart, and we showed that ASK1 inhibition with an inhibitor administered in a clinically relevant fashion at reperfusion reduces infarct sizes, LV wall motion abnormalities, and systolic dysfunction.
